# Supplementary material for: A Chitosan-Based Biomaterial Combined with Mesenchymal Stem Cell-Conditioned Medium for Wound Healing and Skin Regeneration
Source: Int J Mol Sci. 2023 Nov 8;24(22):16080. doi: 10.3390/ijms242216080 (PMC10671656; doi:10.3390/ijms242216080)
Supplement: Supplementary file 1 [file ijms-24-16080-s001.zip › ijms-2685593-supplementary.pdf]

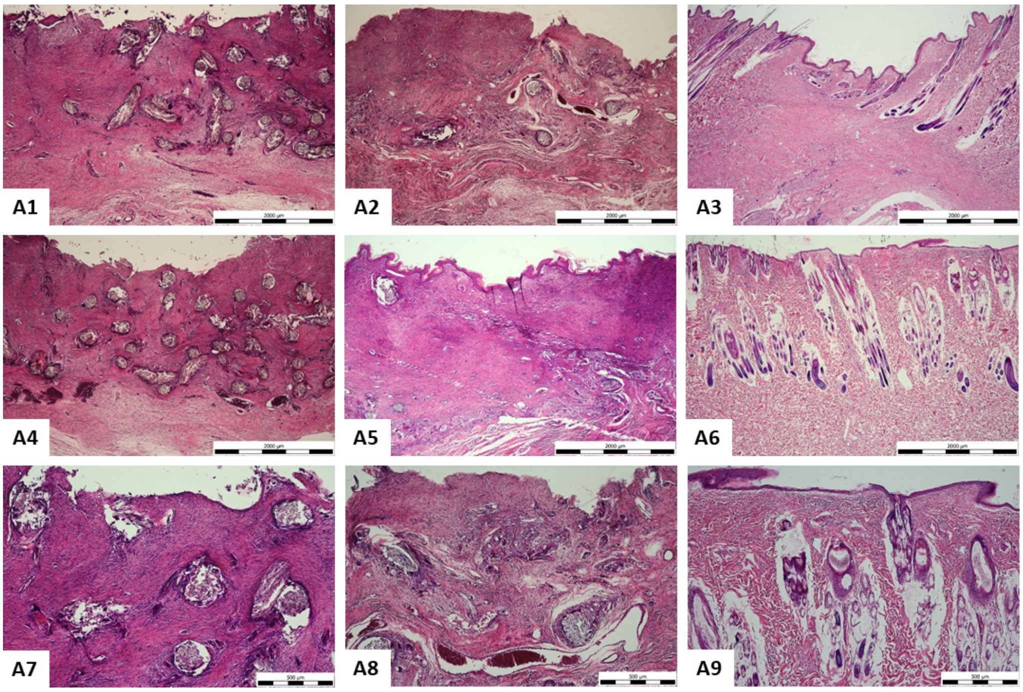

Figure S1. Representative microphotographs of histopathological analysis for control group (A1-A9). Healing periods: 8 weeks (A1, A4, A7), 10 weeks (A2, A5, A8), 12 weeks (A3, A6, A9); A1 – A6 Scale bar = 2000 µm, A7 – A9 Scale bar = 500 µm.

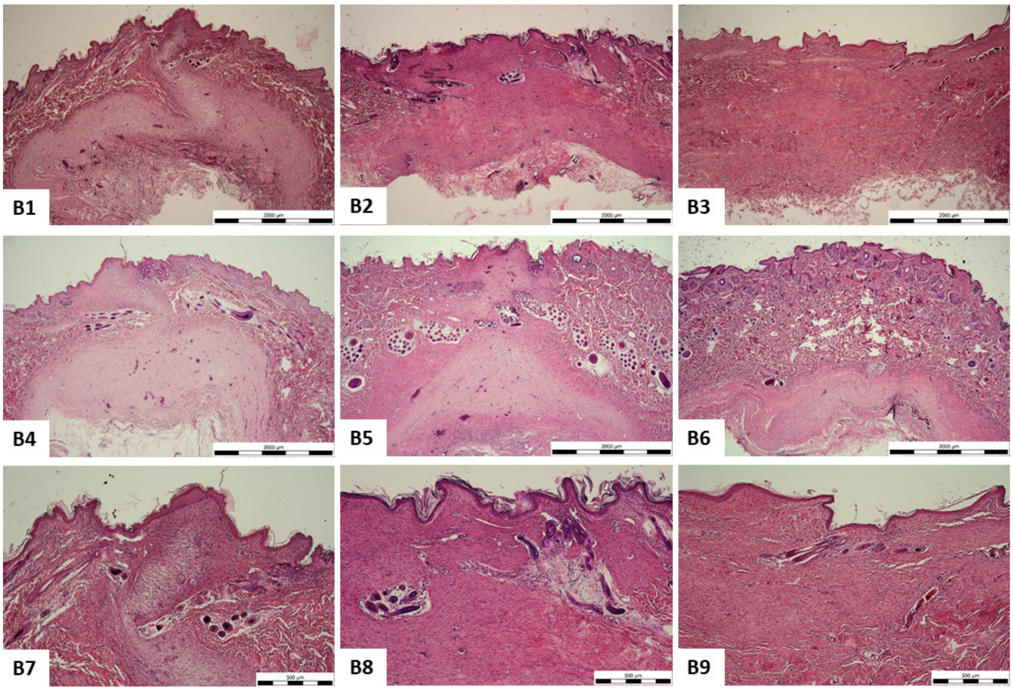

Figure S2. Representative microphotographs of histopathological analysis for implant group (B1-B9). Healing periods: 8 weeks (B1, B4, B7), 10 weeks (B2, B5, B8), 12 weeks (B3, B6, B9); B1 – B6 Scale bar = 2000 µm, A7 – A9 Scale bar = 500 µm.

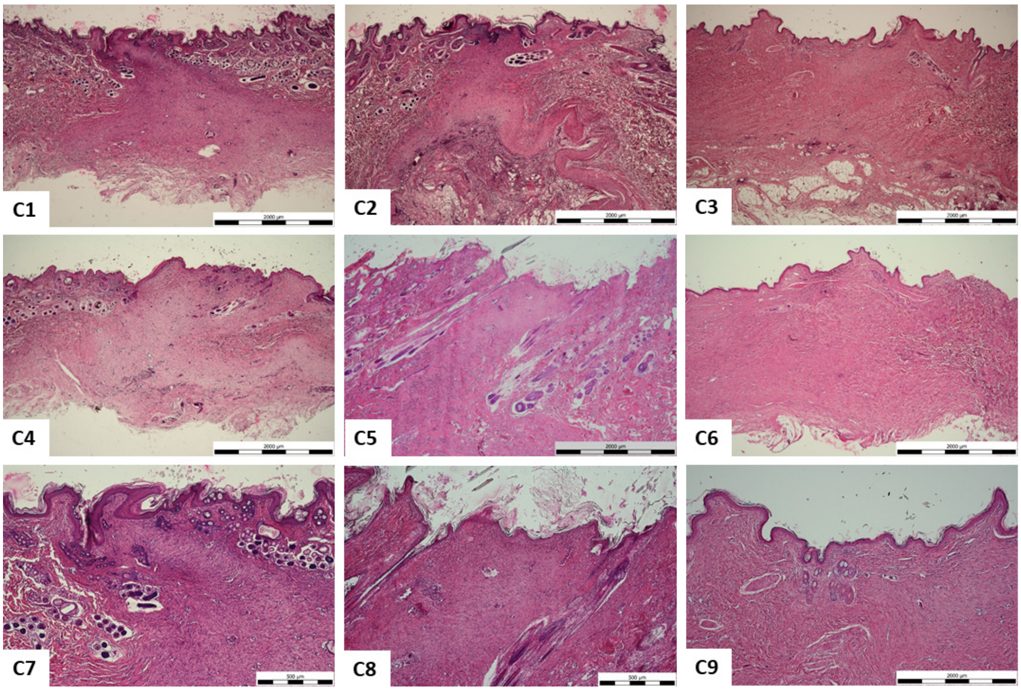

Figure S3. Representative microphotographs of histopathological analysis for implant + medium group (C1-C9). Healing periods: 8 weeks (C1, C4, C7), 10 weeks (C2, C5, C8), 12 weeks (C3, C6, C9); C1 – C6 Scale bar = 2000 µm, C7 – C9 Scale bar = 500 µm.
